# Investigating the Role of Everolimus in mTOR Inhibition and Autophagy Promotion as a Potential Host-Directed Therapeutic Target in *Mycobacterium tuberculosis* Infection

**DOI:** 10.3390/jcm8020232

**Published:** 2019-02-11

**Authors:** Stephen Cerni, Dylan Shafer, Kimberly To, Vishwanath Venketaraman

**Affiliations:** 1 Department of Basic Medical Sciences, College of Osteopathic Medicine of the Pacific, Western University of Health Sciences, Pomona, CA 91766-1854, USA; stephen.cerni@westernu.edu (S.C.) dylan.shafer@westernu.edu (D.S.); 2 Graduate College of Biomedical Sciences, Western University of Health Sciences, Pomona, CA 91766-1854, USA; kimberly.to@westernu.edu

**Keywords:** *Mycobacterium tuberculosis*, host-directed therapies, immune responses

## Abstract

Tuberculosis (TB) is a serious infectious disease caused by the pathogen *Mycobacterium tuberculosis* (*Mtb*). The current therapy consists of a combination of antibiotics over the course of four months. Current treatment protocols run into problems due to the growing antibiotic resistance of *Mtb* and poor compliance to the multi-drug-resistant TB treatment protocol. New treatments are being investigated that target host intracellular processes that could be effective in fighting *Mtb* infections. Autophagy is an intracellular process that is involved in eliminating cellular debris, as well as intracellular pathogens. Mammalian target of rapamycin (mTOR) is an enzyme involved in inhibiting this pathway. Modulation of mTOR and the autophagy cellular machinery are being investigated as potential therapeutic targets for novel *Mtb* treatments. In this review, we discuss the background of *Mtb* pathogenesis, including its interaction with the innate and adaptive immune systems, the mTOR and autophagy pathways, the interaction of *Mtb* with these pathways, and finally, the drug everolimus, which targets these pathways and is a potential novel therapy for TB treatment.

## 1. Introduction

Tuberculosis (TB) is an ancient infectious disease caused by *Mycobacterium tuberculosis* (*Mtb*) that still plagues the modern world. *Mtb* has survived over 70,000 years, and today actively infects around 10 million people annually and lies latent in 1.7 billion people worldwide (23% of the global population) [1]. Claiming over a million lives a year, TB is the leading cause of death by an infectious agent over human immunodeficiency virus/acquired immunodeficiency syndrome (HIV/AIDS). Additionally, immunocompromised individuals, such as those with HIV and type 2 diabetes (T2DM) are at a greater risk of developing active TB. The common treatment for drug-sensitive pulmonary TB by the World Health Organization (WHO) is the Directly Observed Treatment, Short Course (DOTS). DOTS is comprised of an antibiotic regimen of isoniazid (INH), rifampicin (RIF), pyrazinamide (PZA), and ethambutol (ETH) in the initial phase for 2 months, followed by INH and RIF in the continuation phase for 4 months. DOTS therapy is currently the best curative treatment for TB, but the long duration and potential adverse side-effects cause a high non-compliance/drop-out rate. Patient non-compliance increases the risks for development of drug-resistant TB and contributes to TB’s status as one of the top ten causes of death globally [2]. TB’s continuous threat to public health warrants investigation into more effective treatments.

A relatively new modality of TB treatment is called Host Directed Therapy (HDT). HDT aims to augment the endogenous host immune system to battle TB infection, through the use of pharmacology [3]. One target of interest for HDT in TB treatment is autophagy. Autophagy is an intracellular homeostatic process that degrades damaged cellular components and organelles during times of cellular stress via lysosomal degradation [4]. This process is also part of innate immunity and is involved in eliminating intracellular pathogens. Autophagy is also involved in adaptive immunity and might facilitate antigen presentation, which eventually leads to granuloma formation. *Mtb* is able to hinder the host cells’ ability to complete autophagy, through the modulation of mammalian target of rapamycin (mTOR). Everolimus, a potential HDT, might be able to modulate this effect on mTOR and could be a novel treatment for *Mtb*. Here, we have investigated the role of mTOR in the intracellular autophagy of *Mtb* and its implication as a target for future treatment.

## 2. Autophagy Overview

Autophagy is a homeostatic cellular process that involves removing protein aggregates and damaged organelles via lysosomal degradation. This process is crucial for cells to survive under stressful conditions and involves eliminating unnecessary or damaged elements from the cell [5]. It is also a key process for removing invading pathogens, making it a potential target for directed therapies [4,6]. Autophagy has many different subtypes based on the target of degradation and can be selective (for a particular organelle or pathogen) or non-selective (also referred to as macro autophagy or bulk autophagy). For the purposes of this review, we will focus on xenophagy, which is a type of selective autophagy that specifically targets intracellular pathogens [5]. We will review the general process of autophagy as well as specific autophagic processes as they pertain to *Mtb,* including interactions with the innate and adaptive immune systems. An investigation of the relationship between autophagy and *Mtb* is critical in understanding the potential targets of HDT.

Autophagy begins with the formation of an autophagosome, which is a double-membrane-bound vesicle that contains cytoplasmic material [4]. These autophagosomes are non-degradative until they come in contact with lysosomes, forming an autolysosome, which enables them to degrade their contents [4,7]. The induction of autophagy is complex but involves three main components, the phosphoinositide 3-kinase complex 3 (PI3KC3), Unc-51-like Kinase 1 complex (ULK1), and the autophagy-related protein (ATG) complex [6]. The process of autophagy is inhibited by the mTOR complex [6], which is a focus for potential *Mtb* therapeutics. The specific mechanisms of this interaction will be discussed later in this review. Autophagy is not a single pathway and has many effects, both, with the innate immune system and the adaptive immune system. In this review, three autophagy pathways will be discussed: direct pathogen degradation (also referred to as xenophagy), interaction with the innate immune system, and interactions with the adaptive immune system [7].

**Figure 1:**
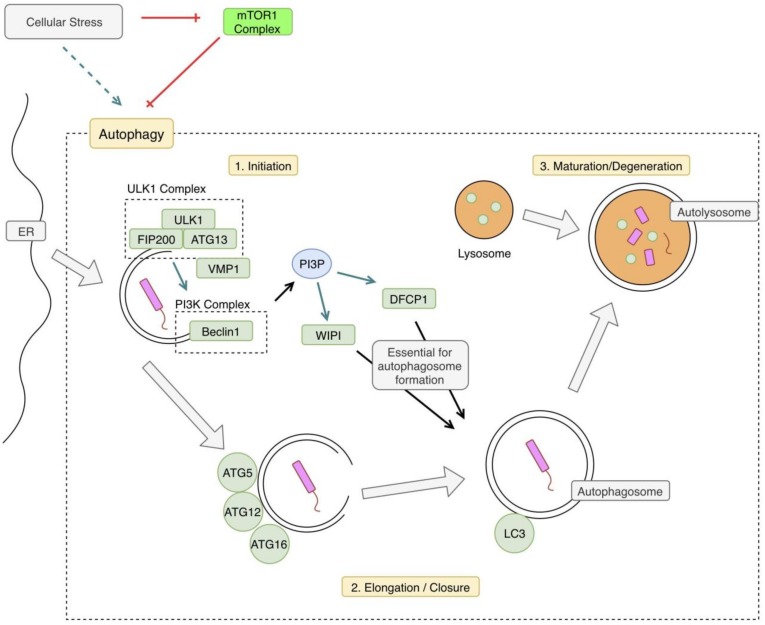
Xenophagy Pathway Overview. Cellular stress including starvation or hypoxia can trigger the autophagy pathway by relieving inhibition by mammalian target of rapamycin 1 (mTOR1). In the case of xenophagy, autophagic mechanisms are triggered by an intracellular pathogen. The pathway begins with phosphorylation of the Unc-51-like Kinase 1 (ULK1) complex, which activates the phosphoinositide 3-kinase complex 3 (PI3KC3) complex. This begins the double-membraned autophagosome formation, which is derived from the endoplasmic reticulum (ER). The next step is elongation and closure of the autophagosome. An autophagy-related protein (ATG) complex comprised of ATG5, ATG12, and ATG16 is involved in this next step. Microtubule-associated protein 1A/1B-light chain 3 (LC3) is also conjugated to the membrane, at this step. Phosphatidylinositol 3 phosphate (PI3P) produced by the PI3KC3 complex is necessary for autophagosome closure, as well. The final step is fusion with a lysosome forming an autolysosome, degrading its contents. VMP1: vacuole membrane protein 1, FIP200: focal adhesion kinase family interacting protein of 200kd, WIPI: WD repeat domain phosphoinositide-interacting protein 1, DFCP1: double FYVE domain-containing protein 1.

Xenophagy is a specific type of autophagy that describes the process of delivering intracellular pathogens to lysosomes via autophagic mechanisms [4]. The precise mechanisms of xenophagy are not well understood; however, there are several proposed hypotheses [7]. An overview of xenophagy can be seen in Figure 1. There are three general steps in the autophagy pathway: initiation, elongation of the autophagosome, and maturation of the autophagolysosome and degradation of its contents.

Before the autophagy mechanisms begin, the intracellular pathogen is tagged with ubiquitin. The process of tagging intracellular bacteria is similar to that of tagging endogenous proteins or organelles for destruction via ubiquitination [6]. Autophagy receptors play an important role in target recognition and delivery to the autophagosome [8]. These specific mechanisms will be discussed in the innate immune system section. The next step is the initiation of autophagosome formation. Generally, autophagy begins with inhibition of mTOR, which results in translocation of the mTOR to the endoplasmic reticulum (ER) and the subsequent phosphorylation of the ULK1 complex, inducing autophagy [9]. This leads to the recruitment of PI3KC3, which produces phosphatidylinositol 3 phosphate (PI3P); this is essential for the autophagosome formation via the double FYVE-containing protein 1 (DFCP1) and the beta transducin (WD)-repeat domain phosphoinositide-interacting (WIPI) proteins [8,10]. Vacuole Membrane Protein 1(VMP1) also appears to play a role in the autophagosome formation, via interaction with beclin 1 and the ULK1 complex [4,8]. The last step of the autophagosome formation requires an ATG12–ATG5 complex and a Microtubule-associated protein 1A/1B-light chain 3 (LC3). The final step of autophagy is the fusion of the autophagosome with the lysosomal compartment [4]. This leads to the formation of the autophagolysosome, which ultimately degrades the pathogen.

In addition to pathogen degradation via xenophagy, autophagy may modulate the innate and adaptive immune systems [4]. With respect to the innate immune system, autophagy can both enhance interferon production and prevent excessive innate immune system activity from being detrimental [4]. With respect to adaptive immunity, autophagy is reportedly involved with both the delivery of exogenous and endogenous antigens to major histocompatibility complex (MHC) class II antigen-presenting molecules and the presentation of viral antigens by MHC class I antigen presenting molecules [4].

## 3. Autophagy and TB in the Innate Immune System

**Figure 2:**
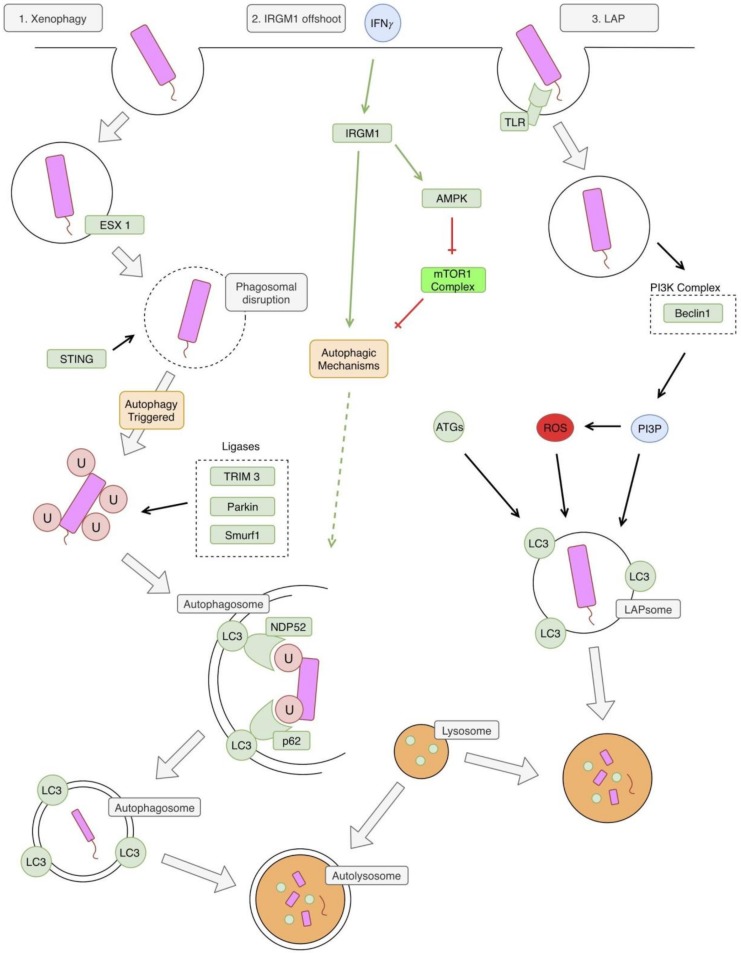
Autophagy and *Mycobacterium tuberculosis* (*Mtb*). The first pathway illustrated in the classic xenophagy pathway. The phagosome is disrupted by bacterial early secretory antigenic target (ESAT)-6 secretion system (ESX1). This allows for recognition of bacterial DNA by the stimulator of interferon genes (STING) pathway, which triggers autophagy. Several ligases are involved in the ubiquitination of the bacteria, including Tripartite motif-containing protein 3 (TRIM3), Parkin, and Smurf1. This ubiquitination facilitates recognition of the bacteria by autophagic mechanisms, leading to the formation of a double-membraned autophagosome. This autophagosome fuses with a lysosome leading to the formation of an autolysosome and the degradation of bacteria. A second pathway, and an offshoot of the first xenophagy pathway, involves immunity-related p47 guanosine triphosphatase family M protein 1 (IRGM1). IRGM1 is triggered by interferon-gamma (IFN-*γ*) and leads to the activation of autophagic mechanisms. The specifics of this pathway are unclear, but it might involve activation of adenosine monophosphate (AMP)-activated protein kinase (AMPK), which relieves the autophagy pathway of its inhibition by mTOR1. The third and final pathway is also referred to as the 1A/1B-light chain 3 (LC3) associated phagocytosis (LAP) pathway. Bacteria is recognized by a toll-like receptor (TLR) and phagocytosed. This process triggers the phosphoinositide 3-kinase (PI3K) complex, leading to the production of PI3P. PI3P leads to the production of reactive oxygen species (ROS), via stabilization of the nicotinamide adenine dinucleotide phosphate hydrogen (NADPH) oxidase-2 (NOX) complex. Phosphatidylinositol 3 phosphate (PI3P), ROS, and other autophagy related proteins (ATGs) lead to the formation of a LAPsome, which is a single-membraned compartment. Microtubule-associated protein 1A/1B-light chain 3 (LC3) is conjugated to the LAPsome, which triggers fusion with a lysosome, leading to degradation of the bacteria. NDP52: nuclear domain 10, protein 2.

There appear to be two autophagy pathways involved in an *Mtb* infection, which ultimately lead to the formation of an autolysosome. These pathways are illustrated in Figure 2. The first classical pathway is xenophagy, which is triggered by a mycobacterial infection via activation of the bacterial early secretory antigenic target (ESAT)-6 secretion system (ESX-1), leading to a disruption of the phagosome membrane [11,12]. The bacterial DNA is next recognized via the stimulator of interferon genes (STING)-dependent pathway which recognizes DNA on the surface of the bacteria [11]. This STING pathway then leads to the direct ubiquitination of the bacteria, which involves activity by the parkin ligase, the Smurf1 ligase, and the TRIM3 ligase [13,14,15,16]. The parkin ligase specifically mediates the linkage of K63 ubiquitin chains [14] and the Smurf1 ligase mediates the linkage of K48 ubiquitin chains [15]. Next, the ubiquitin LC3-binding autophagy adaptors p62 and NPD52 bind ubiquitin, leading to the recruitment of autophagic components, which create a phagophore around the bacteria [11].

An offshoot of this classical pathway involves the murine immunity-related p47 guanosine triphosphatase family M protein 1 (IRGM1). IRGM1 is thought to contribute to this pathway through stabilization of autophagy factors and adenosine monophosphate (AMP)-activated protein kinase (AMPK) [17]. IRGM1 is induced by similar factors that induce general autophagy, including starvation and IFN-*γ* [17].

The second pathway involved is called the LC3 associated phagocytosis (LAP) pathway [13]. This pathway differs from the classical autophagy pathway in that it does not involve a double membrane autophagosome, but rather a single membrane LAPsome [18]. The pathway begins with Toll-like receptor (TLR) activation by *Mtb*, which leads to phagocytosis of the pathogen [19]. The phagocytosed pathogen is now in a single-membrane LAPsome, which has PI3P attached to it (produced by PI3KC3) and includes key LAP proteins, such as Beclin 1 and Rubicon [13]. This leads to the generation of reactive oxygen species (ROS) via the stabilization of the nicotinamide adenine dinucleotide phosphate (NADPH) oxidase-2 (NOX2) complex [20]. The combination of PI3P, ROS, and several other ATGs lead to LC3 being conjugated to the single phagosome membrane [20,21]. This eventually leads to fusion with cellular lysosomes and the destruction of the pathogen [20].

The LAP pathway of autophagy can prevent *Mtb* from inhibiting the maturation of lysosomes. *Mtb* is phagocytosed by an actin-mediated membrane that engulfs the bacterium into a phagosome. Ideally, Rab GTPases activate within the phagosomal membrane to recruit vacuolar ATPases that acidify the phagosomal contents. Then, the phagosome proceeds to fuse with the lysosome which further acidifies and degrades bacteria via enzymatic processes. However, *Mtb* alters the phagosome trafficking pathway through numerous methods, preventing its own elimination by this process [22,23]. *Mtb* uses phosphatidylinositol mannoside (PIM) to stimulate Rab14, promoting phagosome fusion with an early endosome, resulting in the prevention of phagosomal maturation and acidification [24]. Other studies have found *Mtb* uses Lipoarabinomannan Mannosylated (ManLAM) to interfere with the calmodulin complex formation with PI3KC3 and the production of PI3P, which is responsible for the recruitment of vacuolar GTPases to the phagosome, preventing its maturation and fusion with the lysosome [25]. *Mtb* also prevents phagosomal maturation by preventing the phagosome from acquiring Rab5 due to the presence of tryptophan aspartate coat protein (TACO). Through these processes *Mtb* resides in the phagosome at a pH of 6.2 rather than the normal physiologic levels, which can reach a pH of <5.0 [26]. However, when autophagy is induced stronger PI3KC3 and PI3P activation occurs and *Mtb’s* inhibition of lysosomal maturation is overcome [27].

## 4. Autophagy and TB in the Adaptive Immune System

An adaptive cellular immune response is needed to effectively control an *Mtb* infection. After the initial innate immune responses, adaptive immunity develops to control the dividing bacteria. Development of adaptive immune responses occur between 3 to 8 weeks after the initial exposure to *Mtb*. Impaired adaptive immunity often results in clinical TB. Effective host immune responses against *Mtb* infection are dependent on the optimal interactions between the appropriate T cell subsets and infected macrophages.

### 4.1. Type 1 T helper (TH1)

Immunity to *Mtb* infection is associated with the emergence of protective CD4+ T cells that secrete cytokines, resulting in the activation of macrophages and recruitment of monocytes for granuloma formation. Studies in human and animals have demonstrated that acquired immunity to *Mtb* involved multiple T cell subsets with a dominant role of CD4+ T helper cells and aid from the CD8+ T cells [28]. Type 1 T helper (Th1) cells produce interferon-gamma (IFN-*γ*), interleukin, (IL)-2, and tumor necrosis factor (TNF)-beta, which activate macrophages and are responsible for cell-mediated immunity and phagocyte-dependent protective responses. These CD4+ Th1 cells may recognize mycobacterial fragments by the presence of MHC II class molecules on the antigen-presenting cells, such as macrophages, although this concept is debated in recent literature as *Mtb* may have some mechanisms for inhibiting MHC II presentation [29]. Mice with deleted genes for CD4+ or MHC class II molecules are significantly susceptible to *Mtb* infection, strongly establishing the central protective role of the CD4+ T cells [30,31]. Additionally, loss of the CD4+ cell number and function, during the advanced stages of HIV infection, results in progressive primary infection, reactivation of endogenous *Mtb*, and increased susceptibility to re-infection [32].

Following phagocytosis of *Mtb* by macrophages and dendritic cells, IL-12 secretion is induced, driving the development of a Th1 response with the production of IFN-*γ*. IFN-*γ* is involved in the recruitment of T-cells, in the induction of expression of the MHC class II molecules, in the augmentation of antigen presenting cells (APCs), and in the control of *Mtb* growth [33]. Additionally, IFN-*γ* promotes cellular proliferation, cell adhesion, apoptosis, and autophagy [34]. IL-12 is crucial for generation of a protective immunity, with its main function being the induction of expression of IFN-*γ* and activation of antigen-specific lymphocytes. Mice with deleted IL-12p40 gene were more susceptible to infection, had increased bacterial burden, and decreased survival time compared to control mice [35]. IFN-*γ* has been established as the principal mediator for a protective immune response to *Mtb* infection. IFN-*γ* knockout (GKO) mice formed defective granulomas and failed to produce nitrogen intermediates [36]. CD8+ cells also secrete IFN-*γ* but to a lesser extent than that of the CD4+ T cells [34].

### 4.2. Granuloma Formation

Granuloma formation is the hallmark immunopathology of TB, providing a microenvironment for the T cell activation of infected macrophages, to the inhibition of bacterial growth, and localization of the inflammatory and immune responses to the site of infection. Granuloma formation is largely dependent on T cell-mediated immune responses and macrophage-derived cytokines, such as IFN-*γ* and members of the TNF superfamily [37]. Immediate and sustained secretion of chemokines is essential for the recruitment, migration, and aggregation of monocytes and lymphocytes to form granulomas at the sites of *Mtb* infection [38]. Granulomas are composed of various immune cells, including macrophages, dendritic cells, T cell, fibroblasts, epithelioid histiocytes, giant cells, and natural killer cells [37]. The granuloma provides a physical barrier which encapsulates and prevents bacteria from spreading. This local environment allows these immune cells to interact and to effectively kill *Mtb*, which is achieved by macrophage activation and creating an oxygen and nutrient-deprived environment [39]. Critical to granuloma formation is tumor necrosis factor-alpha (TNF-𝛼). Mice deficient in TNF-𝛼 or the 55 kDa TNF receptor, died a rapid death, and with a sustainably higher bacterial burden, compared to control mice [40]. Another study showed structural deficiencies in granulation formation in the TNF-𝛼 gene-targeted mice. Therefore, TNF-𝛼 has a central role in anti-TB immunity, through generation of structurally effective granulomatous response [41].

The failure of the macrophages to acquire mycobactericidal function is likely is due to the host’s inability to generate a sufficient Th1 cell-mediated response. Individuals with compromised cell-mediated immunity, such as HIV-positive patients and diabetic patients, are highly susceptible to *Mtb* infection. A mechanism of immunosuppression is attributed to decreased levels of GSH, which has been shown in HIV and in individuals with T2DM [42,43]. Supplementation with GSH can help restore cytokine balance and enhanced granulomatous response [42,43,44]. GSH is an essential component of intracellular antioxidant systems and functions in the protection of cells against oxidative stress and in maintaining redox homeostasis [45]. GSH could be a potential adjunct therapy to antibiotics and new host-directed therapies in helping relieve oxidative stress in cells. 

### 4.3. Autophagy and Adaptive Immunity

Autophagy contributes to the crosstalk between the innate and adaptive immune response in *Mtb* infection by enhancing antigen presentation. Autophagy is reportedly involved in both the delivery of exogenous and endogenous antigens to MHC class II antigen presenting molecules, along with the presentation of viral antigens by MHC class I antigen presenting molecules [46]. Various authors have shown how autophagy can contribute to the MHC class II presentation, which is particularly important in *Mtb* defense [47,48]. The antigenic contents of the autophagosomes are degraded when they fuse with lysosomes. Then, within the multi-vesicular MHC-II loading compartments (MIICs), the antigenic peptides are fashioned into MHC-II binding groves by the HLA-DM. Autophagy increases the MIIC turnover and strongly improves the MHC class II presentation to CD4+ T cells [7]. A study conducted by Jagannath C. et al., 2009 showed autophagy augmented the efficacy of the BCG vaccine in mice, by improving antigen presentation by antigen presenting cells [49].

In addition to antigen presentation, the adaptive immune system and autophagy maintain a synergistic relationship through the production of cytokines in the defense of *Mtb*. IFN-*γ* produced by Th1 cells induces autophagy [28,29]. In turn, autophagy has been shown to increase the production of TNF-𝛼, IL-6, and IL-8 [7]. In mice, IFN-*γ* produced by Th1 cells promotes the expression of a p47 resistance GTPase, called IFN-*γ*-inducible protein (LRG-47) [30,31]. It is suspected that LRG-47 prompts the creation of autophagolysosomes in the defense of *Mtb* [30]. Multiple experiments have shown that inhibition of certain ATGs, like Beclin-1, and treatment with autophagy inhibitor 3-MA in both murine and human models showed reduction of TNF-𝛼, IL-6, and IL-8 [32]. Each of these cytokines play an important role in the inflammatory response against *Mtb*: IL-8 helps recruit neutrophils, IL-6 stimulates production of acute phase reaction, and TNF-𝛼 is essential in the production of granuloma formation. These findings point towards autophagy as a potent regulator of host defense against *Mtb*.

## 5. mTOR

**Figure 3:**
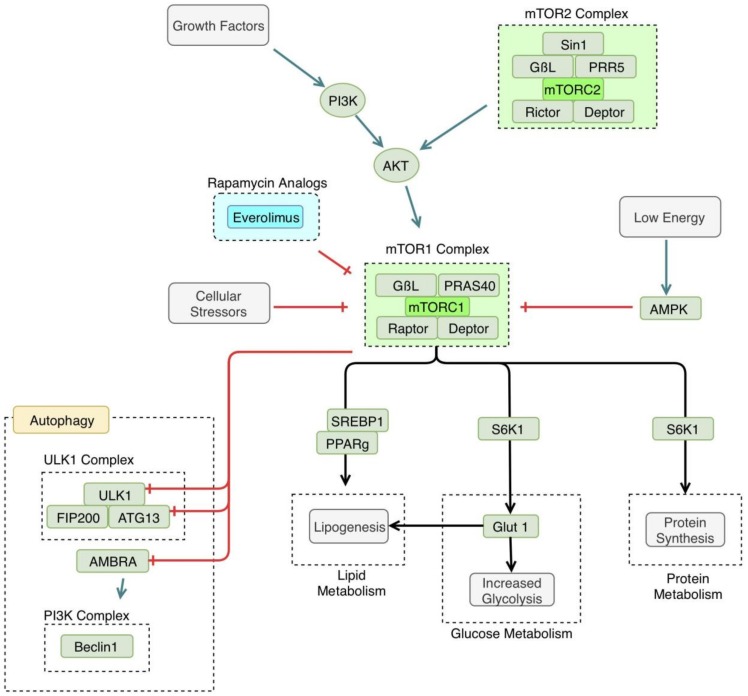
Mammalian target of rapamycin (mTOR) pathway. mTOR1 is triggered by certain growth factors and is generally anabolic, making it an important enzyme in both cancer and processes related to autophagy. Cellular stressors, such as hypoxia or cellular starvation, lead to the inhibition of mTOR, thus, activating the autophagy pathway. mTOR1 inhibits specific enzyme subunits in the autophagy pathway, including Unc-51-like Kinase 1 complex (ULK1), autophagy related protein 13 (ATG13), and activating molecule in Beclin 1-regulated autophagy protein 1 (AMBRA). mTOR1 activation has several metabolic downstream effects, including increased lipogenesis, increased glycolysis, and increased protein synthesis. mTOR is inhibited by compounds in the rapamycin pathway, including everolimus. PI3K: phosphoinositide 3-kinase, AKT: Protein kinase B, PRAS 40: proline-rich AKT substrate of 40kd, PRR5: proline-rich protein 5, AMPK: adenosine monophosphate (AMP)-activated protein kinase, SREBP1: sterol regulatory element-binding protein 1, PPAR *γ*: peroxisome proliferatory-activated receptor *γ*, GLUT1: glucose transporter 1, FIP200: focal adhesion kinase family interacting protein of 200 kd.

The mammalian target of rapamycin (mTOR) is a regulator of many cellular processes involved in growth and differentiation. It is involved in many anabolic pathways and blocks catabolic processes, such as autophagy [50]. mTOR is active when nutrients are readily available to the cell, and is inactivated during times of starvation, leading to the induction of autophagy, which helps the cell survive under these unfavorable conditions [51]. The pathway discussed in this review is also referred to as the Protein kinase B (AKT)/mTOR pathway; illustrated in Figure 3.

mTOR’s specific interaction with autophagic mechanisms has been clarified recently. mTOR interacts with the ULK complex, consisting of the ULK1, FIP200, and ATG13 [52]. mTOR phosphorylates ATG 13, inhibiting the function of the ULK complex [53]. There has also been another proposed mechanism of mTOR autophagy regulation that involves the beclin1 complex. mTOR may inhibit activating molecule in Beclin 1-regulated autophagy protein (AMBRA1), a component of the beclin1 complex [54]. AMBRA1 activity is thought to enhance the ULK1 complex kinase activity [54]. In this way, autophagy is regulated at several points by mTOR, in response to cellular energy demands.

As stated earlier, autophagy is induced by cellular stressors, such as starvation. In a low nutrient or hypoxic environment, mTOR is inactivated, leading to the induction of autophagy [51]. In addition to inhibiting autophagy, mTOR leads to the activation of many metabolic processes, such as glucose metabolism and protein and lipid synthesis [6]. Namely, mTORC1 and mTORC2 increase glycolysis and increase glucose transporter 1 (GLUT1) expression [55]. mTOR is also thought to regulate lipogenesis, via regulation of the activity of peroxisome proliferatory-activated receptor (PPAR) *γ* [56].

## 6. mTOR in TB

mTOR’s activity can be modulated by *Mtb* infection [57]. *Mtb* increases mTOR activity as measured by increased activity in downstream mTOR targets [57]. This is thought to be why there is an increase in cellular aerobic glycolysis [57]. This increase in glucose metabolism is also thought to be a key step in mounting a sufficient immune response against *Mtb* [57]. This metabolic shift during a *Mtb* infection is similar to that of the Warburg effect in cancer cells [58]. The Warburg effect occurs when cancer cells preferentially metabolize glucose by glycolysis, producing lactate, despite having adequate oxygen to undergo oxidative phosphorylation [59].

Another defense mechanism that could be effective in fighting an *Mtb* infection, is the induction of autophagy in granulomas. As discussed earlier, granulomas are a key process in walling off and fighting an *Mtb* infection, due to their ability to foster a beneficial environment for immune cells, as well as provide a physical barrier that prevents the infection from spreading. Additionally, however, granulomas may also provide an environment that fosters autophagic mechanisms. The hypoxic environment in specific types of granulomas has been shown to inhibit mTOR, inducing autophagy, as measured by increased levels of key autophagic enzymes [60].

## 7. Treatment

Several promising HDT strategies exist for the fight against *Mtb*. Strategies that specifically enhance autophagy can be divided into two categories—those involved in inhibiting the mTOR pathway and those that are not. Several points in the AKT/mTOR pathway might be targeted to promote autophagy, such as mTOR itself or AMPK. Direct inhibition of the mTOR complex by rapamycin and its analogs, also referred to as “rapalogs,” is a well-established mechanism to promote autophagy [61]. Activation of AMPK by Metformin also promotes autophagy, via inhibition of mTOR1 (although its role in *Mtb* therapy needs further research) [62]. Drugs that inhibit ATGs and PIK3C3 are also being developed, although inhibition of these enzymes may not completely stop autophagy from occurring [4].

There are also several mTOR-independent targets which can promote autophagy. Ca+ channel blockers, such as Clonidine and Minoxidil, have been found to induce autophagy by increasing the levels of LC3 [63]. Several antipsychotics, such as lithium and valproic acid, also act to increase autophagy by decreasing levels of myo-inositol-1,4,5-triphosphate (IP3), which is thought to promote autophagy, although this mechanism is poorly understood [64]. Lithium has been shown to inhibit the growth of other mycobacterium species, but further studies need to be performed to establish its role in the pathogenesis of *Mtb* [65]. Numerous other drugs have proposed autophagy inducing mechanisms, such as resveratrol, spermidine, EGFR antagonists, vitamin D, and drugs that affect Beclin1 or nitrous oxide (NO) [3,4], although more investigation needs to be done on their therapeutic effect, specifically for *Mtb*. Further discussion of these drugs is outside of the scope of this review, but it is worth mentioning the numerous potential targets of HDT in the search of better, more effective treatments of *Mtb*.

This review will focus specifically on mTOR inhibitors as a potential therapeutic for *Mtb* treatment. Inhibition of mTOR by rapamycin analogs promotes autophagy, increasing the macrophages’ ability to fight Mtb infection. Rapamycin analogs include sirolimus, temsirolimus, and everolimus [61]. These drugs have traditionally been used as anti-cancer treatments, due to their growth-suppressing effects, however, they are being investigated for the treatment of TB, due to their effect on the AKT/mTOR pathway and their autophagy. Of these three drugs, everolimus presents as a good candidate for further investigation as therapy for *Mtb* infection. Everolimus is a novel inhibitor of mTOR that could potentially be used as a therapy for *Mtb* infection and has been shown to decrease *Mtb* growth [6]. Everolimus is administered as an oral tablet and has a lower side effect profile than its injectable counterpart temsirolimus [66]. Additionally, everolimus has a greater bioavailability than sirolimus and it decreases vascular inflammation, more so than sirolimus [67].

The beneficial effects of everolimus on autophagy must be carefully weighed against its effects on the immune system, when treating *Mtb*. At high doses, everolimus is an effective immunosuppressant and is FDA approved for organ transplant recipients and cancer patients, but at lower doses it has shown to have an augmentative effect on host immune response [68,69,70]. A study conducted in 2014 showed that a group of healthy elderly individuals, treated with everolimus, showed a 20% improvement in their protective response after an influenza vaccination. This response included the reduced expression of programmed cell death-1 receptor on CD8+ and CD4+ T-cells via the inhibition of mTOR [69]. The subjects in this study were treated at a lower dose of everolimus than the transplant patients who are conversely at an increased risk of *Mtb* infection when using everolimus [6]. A proposed mechanism for delivery of everolimus to target cells infected with *Mtb*, without causing systemic immunosuppression, is through an inhaled nanoparticle preparation [71]. An in vitro study of inhalable rapamycin showed a more effective intracellular clearing of mycobacterium than rapamycin in solution [72]. These findings suggest that rapalogs, such as everolimus, might have a promising future as an HDT against TB.

## 8. Conclusions

*Mtb* infections pose a major global public health threat. Current treatments still revolve around antibiotic DOTS therapy. As antibiotic resistance grows other therapeutic targets will become more and more essential. HDT is a novel treatment strategy, aimed at using host immune mechanisms to battle infection. In this paper, we investigated the current literature on the AKT/mTOR pathway and autophagy, and their role in the pathogenesis of *Mtb*. We also investigated the current literature on everolimus as a novel therapy for *Mtb* infection, modulating cellular autophagic mechanisms via the inhibition of mTOR. The benefits of everolimus include less dependence on the use of DOTS therapy and the growing threat of resistant *Mtb*. These benefits must be carefully weighed against the immunosuppressive effect of everolimus. Novel drug delivery systems, such as inhaled nanoparticles might address this, although understanding the risks of treatment with each individual patient must also be carefully considered. Continued investigation of these novel therapeutic targets is crucial to addressing the global threat of *Mtb*.
